# Outer Membrane Porins Contribute to Antimicrobial Resistance in Gram-Negative Bacteria

**DOI:** 10.3390/microorganisms11071690

**Published:** 2023-06-28

**Authors:** Gang Zhou, Qian Wang, Yingsi Wang, Xia Wen, Hong Peng, Ruqun Peng, Qingshan Shi, Xiaobao Xie, Liangqiu Li

**Affiliations:** Key Laboratory of Agricultural Microbiomics and Precision Application (MARA), Guangdong Provincial Key Laboratory of Microbial Culture Collection and Application, Key Laboratory of Agricultural Microbiome (MARA), State Key Laboratory of Applied Microbiology Southern China, Institute of Microbiology, Guangdong Academy of Sciences, Guangzhou 510070, China; zgbees@gdim.cn (G.Z.); wangqian@gdim.cn (Q.W.); wongvincy@163.com (Y.W.); wexi153589@163.com (X.W.); tangph163@163.com (H.P.); pengruqun@gdim.cn (R.P.); shiqingshan@hotmail.com (Q.S.); xiexb@gdim.cn (X.X.)

**Keywords:** gram-negative bacteria, outer membrane proteins, permeability properties, resistant mechanisms, multidrug-resistant bacteria

## Abstract

Gram-negative bacteria depend on their cell membranes for survival and environmental adaptation. They contain two membranes, one of which is the outer membrane (OM), which is home to several different outer membrane proteins (Omps). One class of important Omps is porins, which mediate the inflow of nutrients and several antimicrobial drugs. The microorganism’s sensitivity to antibiotics, which are predominantly targeted at internal sites, is greatly influenced by the permeability characteristics of porins. In this review, the properties and interactions of five common porins, OmpA, OmpC, OmpF, OmpW, and OmpX, in connection to porin-mediated permeability are outlined. Meanwhile, this review also highlighted the discovered regulatory characteristics and identified molecular mechanisms in antibiotic penetration through porins. Taken together, uncovering porins’ functional properties will pave the way to investigate effective agents or approaches that use porins as targets to get rid of resistant gram-negative bacteria.

## 1. Introduction

The cell membranes of gram-negative bacteria are crucial for their survival and environmental adaptation. Generally, these membranes not only give gram-negative bacteria surface specificity but also operate as a functional and protective barrier for them [1]. Gram-negative bacteria have double membranes that include an outer membrane (OM) made up of outer membrane proteins (Omps) and other components [2,3]. In some circumstances, toxic substances, such as antibiotics, are prevented from transiting through the OM [4,5], creating a considerable obstacle to the eradication of gram-negative infections with traditional or novel antimicrobial agents.

Porins, a subclass of transmembrane pore-forming Omps, create tiny channels in the membrane and allow passive transport of hydrophilic compounds, which helps to modulate cellular permeability and increase antibiotic resistance [5,6,7,8]. In gram-negative bacteria, porins are the most prevalent Omps of the OM, and they can be divided into two groups based on how they function: non-specific porins and specific porins [5,9]. Porins have been studied in a variety of bacteria, including *Acinetobacter baumannii* [10], *Escherichia coli* [11,12], *Neisseria gonorrhoeae* [13,14], and *Klebsiella pneumoniae* [15,16]. Porins appear to play a role in the envelope integrity of gram-negative bacteria in addition to their role in the passive transport of a range of chemicals. For example, the non-specific porin of outer membrane protein A (OmpA) promotes the passive transport of numerous tiny molecules [17,18]. Additionally, OmpA also has a flexible periplasmic domain that interacts non-covalently with peptidoglycan [19], supporting the integrity of the envelope. Omps are usually encased by an annular shell of asymmetric lipids, which mediates higher-order Omp–lipid–Omp complexes with adjacent Omps, and the basic unit of supramolecular Omp assembly generally extends across the entire cell surface, coupling the OM’s required multifunctionality to its stability and impermeability [20].

An alarming global problem has emerged in the form of rising antimicrobial resistance (AMR), which is brought on by improper and excessive usage of antimicrobials as well as a shortage of novel and creative antibiotics in development [21]. AMR is one of the top ten worldwide public health hazards, according to studies from the World Health Organization (WHO). The financial burden of AMR on the world is steadily increasing [1]. In order to battle gram-negative bacteria carrying AMR traits, new drug resistance elimination techniques will need to be developed based on a thorough understanding of the structural properties, functions, and regulatory mechanisms of porins. For instance, it was discovered that a novel diazabicyclooctenone beta-lactamase inhibitor inhibits key carbapenemases classes and is OmpA-dependent, which in turn improves sulbactam activity [18]. To eradicate MDR strains of *A. baumannii*, OmpA blockers can work in tandem with last-resort antibiotics, such as colistin [22,23]. In addition, the results of AutoDockTools and Schrodinger’s QikProp suggest that bioactive seaweed-sulfated polysaccharides (SSPs) can be a promising therapeutic choice for extensively drug-resistant *Salmonella typhi* targeting OmpF [24].

Although porins participate in multiple physiological processes, including the uptake of small molecules for growth and cell function, inflammation and immune response of the host, and cell–cell contacts [25], this review focuses on providing a comprehensive overview of the properties and functions of OmpA, OmpC, OmpF, OmpW, and OmpX in relation to drug permeability and membrane integrity. Additionally, this review also investigates the interrelationships among these porins and the underlying resistance mechanisms they mediate. A thorough understanding of the functions and characteristics of diverse porins would prove advantageous in the development of antibiotics with enhanced permeability and the management of gram-negative bacterial resistance.

## 2. OmpA

OmpA, a monomeric protein with a β-barrel shape that promotes the diffusion of negatively charged β-lactam antibiotics, has been demonstrated to be involved in drug resistance [10,26,27]. Furthermore, OmpA interacts with the bacterial cell wall to attach the outer membrane, and its C-terminal periplasmic domain non-covalently binds to the peptidoglycan layer via two conservative amino acids in OmpA, aspartate at position 271 and arginine at position 286 [28]. In addition, the periplasmic gap formed by disulfide linkages between OmpA’s β strands 4 and 5 in the N- and C-terminal fragments can increase the protein’s assembly efficiency in *E. coli* when the cysteine residues are aligned in the completely folded β-barrel [29]. The alleles of *ompA* were observed to have a considerable effect on cell surface hydrophobicity and charge in *E. coli*, which are important in stress response [30].

OmpA is a significant non-specific channel that helps various bacteria maintain the integrity of their membranes. In comparison to the wild type (WT) strain, an *ompA* mutant of *A. baumannii* showed a 2- to 3-fold reduced permeability to cephalothin/cephaloridine, and the minimal inhibitory concentrations (MICs) of nalidixic acid, chloramphenicol, trimethoprim, aztreonam, imipenem, colistin, and meropenem also decreased in the mutant [31,32,33,34,35]. OmpA’s impact on maintaining membrane integrity helps to explain these results because compromised membrane integrity can increase the intracellular diffusion of antibiotics [31,35]. The primary surface glycoproteins, Pgm6 and Pgm7, in *Porphyromonas gingivalis* have been demonstrated to cause resistance to the bactericidal activity of human cathelicidin LL-37 and are known as OmpA-like proteins [36]. The *ompA* mutant of *E. coli* was more sensitive to a variety of antibiotics, including β-lactams, amphenicols, glycopeptides, and lincosamides, compared to the WT [6]. Furthermore, our team discovered that *Citrobacter werkmanii* showed increased 1,2-benzisothiazolin-3-one (BIT) resistance following the inactivation of *ompA* [37]. By preserving the stability of the outer membrane, OmpA also defends *Salmonella* Typhimurium from the two β-lactam antibiotics: ceftazidime and meropenem [38].

The flexible C-terminal domain of OmpA interacts non-covalently with peptidoglycan, which is critical for the maintenance of cell wall integrity [19]. OmpA’s C-terminal domains are entirely in charge of how this protein affects antibiotic resistance. The mutant strain of *ompA1C*, which has the C-terminal domain of OmpA chromosomally deleted, displayed a nearly identical phenotype to the *ompA* mutant of *E. coli* in antibiotic sensitivity [6]. The turgor of the bacterial envelope degrades in the absence of peptidoglycan interaction, increasing membrane permeability and antibiotic penetration [6]. In *A. baumannii*, OmpA typically serves as the main non-specific slow porin, along with β-lactamases and multidrug efflux pumps, such as AdeABC and AdeIJK, to exhibit high levels of intrinsic antibiotic resistance [39]. In addition, OmpA also connects to peptidoglycan (PG) from *A. baumannii* via its C-terminal region, where Asp271 and Arg286 link to the peptidoglycan’s diaminopimelic acid [40]. This interaction could regulate how bacteria produce outer membrane vesicles (OMVs) and preserve the stability of the membrane [41]. In order to combat antibiotic resistance, OMVs with OmpA in their membrane aggressively drain extracellular drugs [42,43,44].

**Figure 1:**
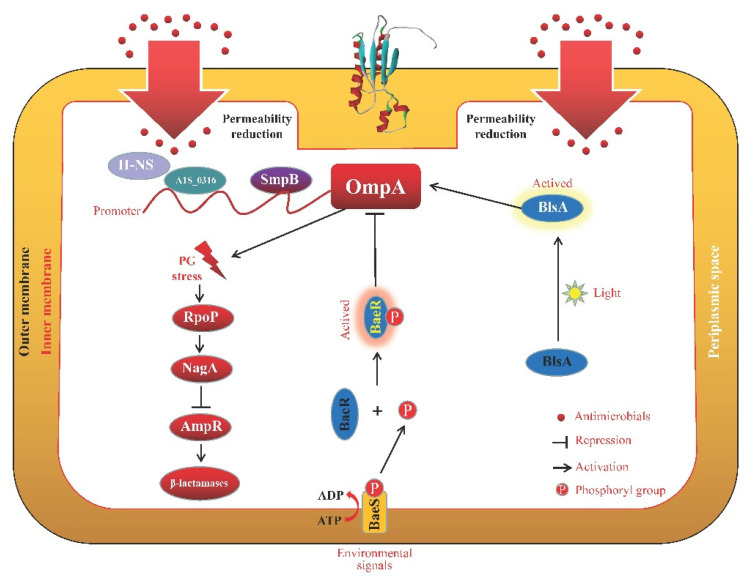
Schematic representation of the OmpA regulation pathways. The promoter of OmpA could be combined by SmpB [45] and an anti-(H-NS) repressor of A1S_0316 [46]. In *Stenotrophomonas maltophilia*, OmpA participates in the β-lactam resistance response through the sigma (P)-NagA-L1/L2 regulatory circuit [47]. In response to environmental signals, the sensor kinase BaeS undergoes auto-phosphorylation from ATP at a conserved histidine residue. After that, the phosphoryl group is transferred to the BaeR, where it interacts with the receiver domain at a conserved aspartate residue [141]. Phosphorylated BaeR (BaeR-P) inhibits OmpA expression, resulting in lower antimicrobial permeability [135]. The expressional levels of OmpA in *A. baumannii* can be positively regulated by BlsA, which influences the penetration of EtBr and meropenem [48].

The expression of OmpA can be regulated by several genes (Figure 1). In *Aeromonas veronii*, the lower transcription expression of *ompA* could be caused by the deletion of small protein B (SmpB) and four regions (−46 to −28 bp, −18 to +4 bp, +21 to +31 bp, and +48 to +59 bp) of the OmpA promoter combined by SmpB, which suggests that the SmpB protein was positively responsible for controlling OmpA expression at the stationary stage [45]. In *A. baumannii*, A1S_0316 displayed a higher affinity for binding to the OmpA promotor region than the global repressor H-NS, and it operates as an anti-repressor on the OmpA promotor region by preventing the binding of the AbH-NS protein [46]. OmpA in *Stenotrophomonas maltophilia* KJ plays its β-lactam susceptibility response through the sigma (P)-NagA-L1/L2 regulatory circuit, according to transcriptome analysis and real-time quantitative (qRT-PCR) experiments [47]. PG stress, which is generated by the loss of interaction between OmpA and PG layers, triggers the upregulation of RpoP (σ^P^) and the expression of *nagA*. The increased NagA activity encourages the synthesis of repressor ligands, which are believed to partially displace activator ligands from AmpR and reduce the production of ceftazidime-induced β-lactamases [47]. More recently, it has been found that BlsA, a blue light-sensing protein, can impact the expressional levels of the *ompA* gene of *A. baumannii* under light conditions, which affects the efficiency of membrane penetration of lipophilic ethidium bromide (EtBr) and meropenem absorption [48].

## 3. OmpC

Another porin expressed by gram-negative bacteria is OmpC [49]. OmpC is composed of 16-stranded beta barrels with negatively charged amino acids that contribute to the formation of the porin’s eyelet and promote its size-exclusion and permeability properties [50]. OmpC facilitates both the entry and resistance of antibiotics, such as β-lactams, as well as the movement of hydrophilic substances with a low molecular weight over the outer membrane [49,51]. The mutation of OmpC damages structural integrity and alters OM permeability [7].

OmpC is involved in the transfer of antibiotics [52]. A slight rise in imipenem MIC is connected to the decrease or loss of OmpC in clinical isolates of *Enterobacter aerogenes* and *Enterobacter cloacae* [53,54]. The transcription and protein expression of OmpC were decreased in all carbapenem non-susceptible (CP-NS) *E. coli* isolates, and the carbapenem susceptibility of one isolate was restored by cloning the *ompC* gene [55]. OmpC mutants have enhanced cefotaxime resistance in clinical isolates of multidrug-resistant *E. coli* [52]. Meanwhile, the *ompC* deletion mutant of *E. coli* was resistant to streptomycin, fusidic acid, and nitrofurantoin; however, it was susceptible to carbapenems, cefepime, carbapenems, fourth-generation cephalosporins, imipenem, vancomycin, and puromycin [6,56,57].

**Figure 2:**
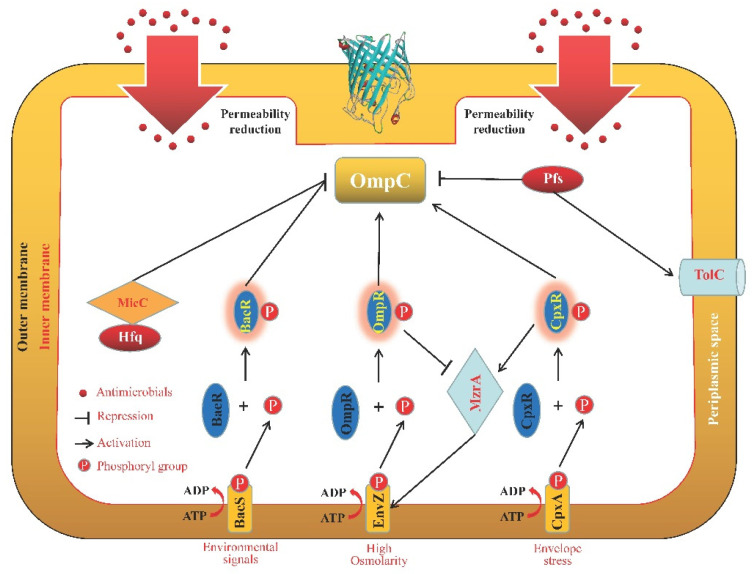
Schematic representation of the OmpC regulation pathways. The expression of OmpC can be inhibited by the two-component regulatory system of BaeSR [135] and MicC in the presence of an Hfq RNA chaperone [61]. When EnvZ detects high osmolarity, a phosphotransfer from EnvZ to OmpR increases the synthesis of phosphorylated OmpR (OmpR-P), which increases the expression of OmpC [59,142]. Meanwhile, the response regulator CpxR receives a phosphoryl group once the sensor kinase CpxA recognizes envelop stress. The expression of OmpC is subsequently increased by the activated CpxR [134]. The activated CpxA/CpxR induces the synthesis of MzrA, which also increases EnvZ/OmpR activity and OmpC expression. A high concentration of OmpR~P downregulates *mzrA*, which forms a negative regulatory feedback cycle [138]. In addition, the transcription levels of OmpC and TolC are negatively and positively regulated by Pfs [139].

One of the key mechanisms contributing to higher MICs for specific antibiotics was mutations in OmpC. Mutation prediction suggests that the primary contributor to carbapenem resistance is amino acid alterations, such as D192G, in the *ompC* of carbapenem-resistant *E. coli* [58]. Additionally, an adjacent region of the OmpC protein in *E. coli* contained a duplication of eight amino acids [57]. Based on specific R66, L67, Y64, F69, M65, and L67 locations, this duplication might be exploited to create salt bridges with the negatively charged residues lining the other side of the barrel wall and changing the pore’s electrostatic field [57]. Moreover, the following factors can be used to illustrate how the identified insertion affected OmpC function: disruption of tertiary or quaternary structure; preventing phage attachment and sterically restricting the movement of molecules; and disruption of the hydrophobicity and charge of porins by limiting their ability to interact with substances, such as antibiotics, to promote transport [57]. In avian pathogenic *E. coli*, quantitative real-time reverse transcription PCR (RT-qPCR) research revealed that EnvZ, the histidine kinase (HK) of OmpR/EnvZ (Figure 2), could affect the expression of biofilms and stress response genes, including *ompC* [59]. Small regulatory RNA (sRNA)-dependent control of gene expression enables cells to quickly and efficiently respond to different growth conditions [60]. Meanwhile, it was discovered that the 109-nucleotide MicC sRNA (Figure 2) suppresses OmpC expression in *E. coli* by directly base-pairing to a 5′ untranslated region of the *ompC* mRNA, which requires the Hfq RNA chaperone to function [61].

## 4. OmpF

Another major porin expressed and extensively distributed in gram-negative bacteria OM is OmpF [62]. OmpF folds as a 16-stranded antiparallel β-barrel in tight homotrimers [63]. OmpF constructs its crystal structure with two asymmetric trimers in the tetragonal form [64].

It has been found that OmpF plays a crucial role in the permeation of short antimicrobial peptides (AMPs) by providing access to the lipopolysaccharide (LPS) binding site [65]. The antibiotic resistance of the *ompF*-deficient mutant indicates that OmpF is commonly the primary pathway by which antibiotics enter the OM [66]. Furthermore, it has been demonstrated that the non-specific porin OmpF of *E. coli* enables β-lactams (such as zwitterionic, ampicillin, and amoxicillin) and fluoroquinolones (such as enrofloxacin and norfloxacin) to penetrate the OM due to their strong affinity to OmpF [6,50,67,68], which partially depends on a two-step kinetic model. The dipolar molecule first generates an MD-ES0 conformation by aligning to the electric field within the OmpF channel before being reoriented into an MD-ES1 conformation for transport [69]. In addition, the electroosmotic flow rather than the electrophoretic force dominates the dynamics of antibiotic capture and transport of norfloxacin, ciprofloxacin, and enoxacin across a voltage-biased OmpF nanopore [70]. The *ompF* mutant was found to be resistant to many β-lactam antibiotics (including ampicillin and cefoxitin) in *E. aerogenes* [71], *Pseudomonas aeruginosa* [72], *E. coli* [6,73,74], *Serratia marcescens* [75], and *K. pneumoniae* [76]. OmpF also contributes to resisting numerous other different classes of antibiotics in addition to β-lactam antibiotics. In *E. coli*, the main route for enrofloxacin’s entrance is OmpF [63], and the reduced expression of OmpF led to the spread of quinolone resistance [77]. Studies on *E. coli* also showed that OmpF expression is activated in response to tetracycline [78,79]. Similarly, *S. marcescens* lacking OmpF had less antibiotic permeability and much higher nitrofurantoin MIC values [80]. Taken together, the *ompF* mutant was shown to be resistant to a wide range of antibiotics from various groups, such as β-lactams, tetracyclines, amphenicols, quinolones licosamides, and steroides [6].

**Figure 3:**
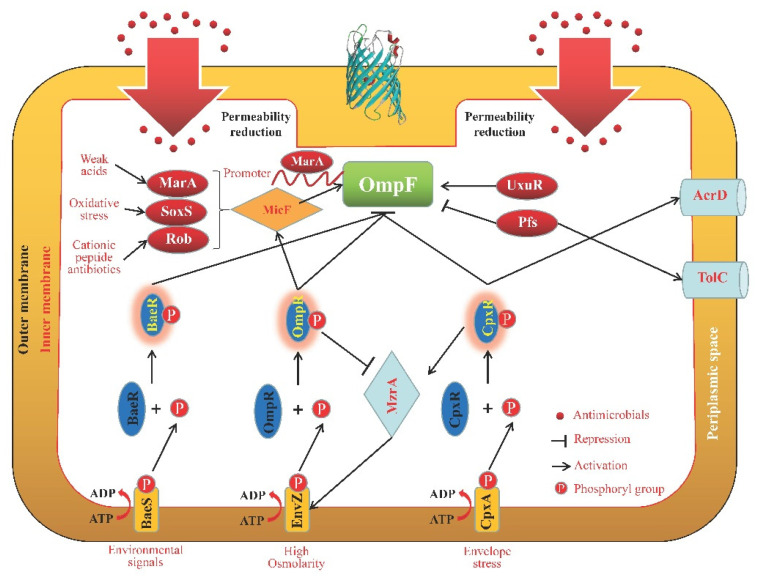
Schematic representation of the OmpF regulation pathways. The two-component system BaeSR, EnvZ/OmpR, and CpxAR negatively control OmpF expression partially through the MicF sRNA at the transcriptional and/or post-transcriptional stages [81,82]. The activation of *micF* can be regulated by OmpR, MarA, SoxS, and Rob [83]. In addition, MarA can directly inhibit OmpF expression at the transcriptional level, or indirectly at the post-transcriptional level by activating MicF [25]. Pfs controls the transcription levels of OmpF and TolC in a negative and positive manner, respectively [139]. UxuR positively regulates OmpF expression with unknown mechanisms [84].

Several works have demonstrated that the OmpF expression can be influenced by various systems or genes (Figure 3). The two-component system EnvZ/OmpR controls OmpF expression in response to nalidixic acid resistance [81]. In this system, activation of the response regulator OmpR leads to phosphorylation, and OmpR~P suppresses OmpF expression both at the transcriptional and post-transcriptional stages, the latter through the MicF sRNA [82]. It is known that the *micF* promoter region is bound by four transcriptional regulators: OmpR, MarA (the key transcriptional regulator encoded by the *marRAB* operon), SoxS, and Rob, which regulate the activation of *micF* expression. The *micF* gene, encoding a non-translated 93 nt antisense RNA, binds its target *ompF* mRNA and regulates *ompF* expression by inhibiting its translation and inducing degradation of the message in *E. coli* [83]. In addition, by binding to a conserved MarA-binding site in the promoter region of *ompF*, the transcriptional regulator of MarA can directly inhibit the expression of OmpF at the transcriptional level, or indirectly at the post-transcriptional level by activating the previously discussed MicF [25]. UxuR belongs to the GntR family of transcriptional regulators. A reduced amount of outer membrane porin OmpF was observed with the deletion of *uxuR* in *E. coli*, which suggests that UxuR regulates OmpF expression with unknown mechanisms [84]. Furthermore, our team discovered that the OmpF upstream promoter in *C. werkmanii* and its transcription could be combined with and negatively regulated by the maltose metabolic regulator MalT for the first time, but we did not investigate how OmpF mutants might react to various antibiotics [85].

## 5. OmpW

An eight-stranded β-barrel with a hydrophobic channel is formed by OmpW, a member of the small Omp family [86,87]. OmpW plays a role in the transport of tiny hydrophobic chemicals, which helps to explain why some antimicrobials are less efficient at inhibiting bacterial growth [86].

In *E. coli* strains resistant to nalidixic acid, OmpW was discovered to be upregulated [81]. However, mass spectrometry and Western blotting results revealed that OmpW was downregulated in kanamycin-resistant *E. coli* K-12 strains, colistin/carbapenem-resistant *A. baumannii* mutants, and ceftriaxone-resistant *S. typhimurium* strains [86,88], and were once more consistent with the fact that porins restrict the entry of β-lactams into cells [89]. According to proteomic research, OmpW in *E. coli* has been associated with bacterial resistance to drugs such as ampicillin, tetracycline, and ceftriaxone [90]. *E. coli* also protects itself from enrofloxacin by reducing OmpW expression by restricting the transport and intracellular concentration of this drug [91]. Thereby, it was discovered that under tobramycin stress, the expression of *ompW* in *A. baumannii* was markedly downregulated [92]. In *A. baumannii* isolates, *ompW* expression increased and decreased in response to ciprofloxacin and imipenem, respectively [93]. 

The knockout of the *ompW* gene also demonstrated that OmpW displayed antimicrobial resistance in many bacteria. OmpW appears to be the receptor or a component of the receptor for colicin S4, chlortetracycline, neomycin, and ampicillin, as evidenced by the resistance of *E. coli* mutants lacking the OmpW to these drugs [94,95,96]. The methyl viologen sensitivity of *ΔompW* of *S. typhimurium* is 2.5 times greater than the WT [97]. The loss of OmpW in *Actinobacillus pleuropneumoniae* affects bacterial susceptibility to penicillin, kanamycin, and polymyxin B [98]. Meanwhile, the *ompW* of *E. coli* is also involved in the ethidium multidrug resistance gene E (*emrE*)-mediated substrate efflux process and is mechanistically connected to EmrE [99].

**Figure 4:**
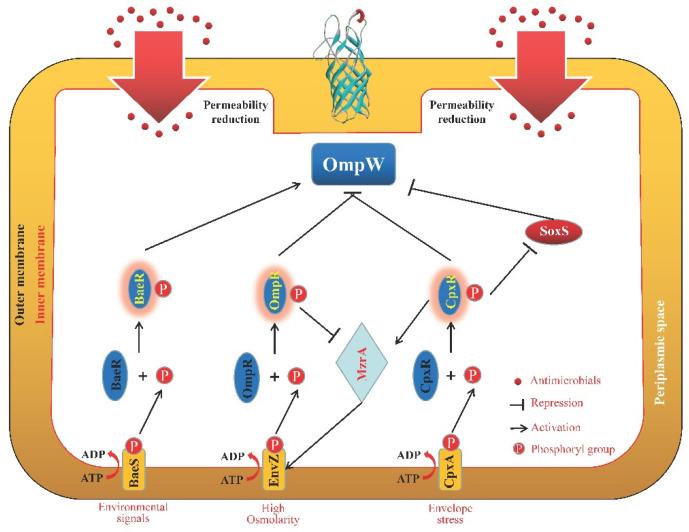
Schematic representation of the OmpW regulation pathways. The two-component system of BaeSR positively regulated OmpW expression [100]. The other two systems, EnvZ/OmpR [102] and CpxAR [134], negatively control OmpW expression. Additionally, SoxS negatively regulated OmpW expression in *E. coli* [101].

As shown in Figure 4, *baeR*, a regulator gene of the BaeSR two-component system, was discovered to affect OmpW expression in *S. typhimurium* [100]. It was discovered that an oxidative stress-related transcriptional SoxS factor negatively regulated OmpW in *E. coli* [101]. When specific environmental signals are detected by EnvZ, a phosphotransfer from EnvZ’s His243 to OmpR’s Asp55 causes an increase in the cellular level of phosphorylated OmpR (OmpR-P), which implies an active state of EnvZ/OmpR. This state changes the OMP composition and leads to differential expression of *ompW* and other OMP genes, increasing resistance to β-lactams, while OmpR directly suppresses *ompW* in *Salmonella enteritidis* [102].

## 6. OmpX

OmpX was first described for *E. cloacae* [103], but its homologs, such as PagC, Lom, Rck, Ail, and y1324, have been found in other gram-negative bacteria, including *S. typhimurium* [104], *E. coli* [105], *E. aerogenes* [106], and *Yersinia pestis* [107]. An OmpX protein precursor with 172 amino acid residues and a 23 amino acid residue N-terminal signal sequence is encoded by the *ompX* gene in *E. cloacae* [103]. According to the X-ray crystallography and NMR structures, OmpX from *E. coli* forms an eight-stranded antiparallel β-barrel in the DHPC micelles [108]. Recently, it was found that OmpX folding is influenced by both the insert length within a set of equivalent loop insertions and its hydrophobic character [109].

Numerous investigations have revealed that *ompX* is crucial for regulating how bacterial strains respond to antimicrobials [106,107]. The loss of *ompX* in a fimbriated strain of *E. coli* PC31 resulted in antibiotic resistance to numerous antibiotics, which was attributed to increased exopolysaccharide production [80]. The deletion of *ompX* in *E. coli* improved resistance to a variety of hydrophobic antibiotics such as amikacin, cephalothin, gentamicin, novobiocin, nalidixic acid, and sulfonamides [80]. Similar to this, our research also discovered that OmpX in *C. werkmanii* regulates resistance to drugs, such as tetracycline, ciprofloxacin, chloramphenicol, lincomycin, rifampicin, aminoglycosides (kanamycin and streptomycin), and β-lactams (ampicillin, carbenicillin, ceftazidime, and imipenem) [110]. Meanwhile, the overexpression of OmpX in *E. aerogenes* results in higher β-lactam resistance, which can be explained by a significant decrease in Omp36 porin [111]. OmpX was found to be 1.7 times more abundant in drug-resistant *S. typhimurium* isolates than in drug-sensitive isolates [112]. These findings imply that the under or overexpression of *ompX* affects hydrophobic chemical transport across the membrane but does not impact substrate preference [80]. However, the lack of *ompX* in *E. cloacae* had no appreciable effect on porin regulation or susceptibility to β-lactam antibiotics [103].

**Figure 5:**
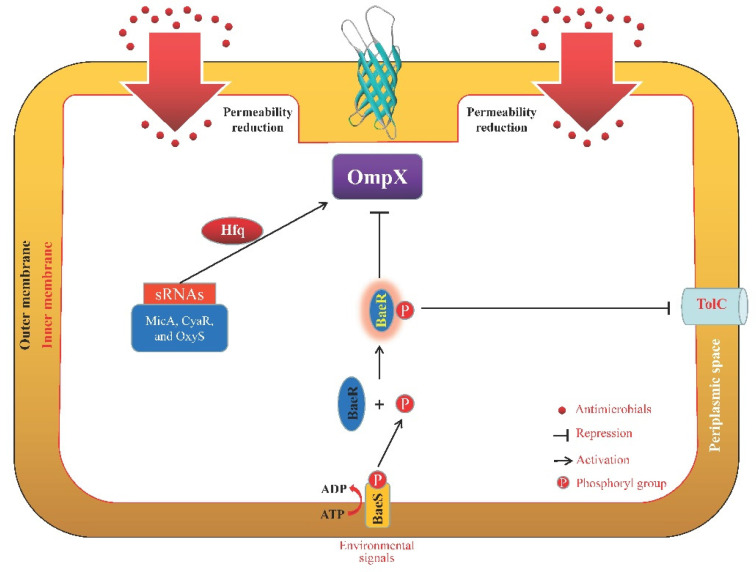
Schematic representation of the OmpX regulation pathways. The two-component system BaeSR negatively regulates OmpX expression, which is linked to a decrease in TolC [135]. In addition, MicA, CyaR, and OxyS were needed for the stability of the *ompX* mRNA in an Hfq-dependent manner [114].

The ceftriaxone resistance functions of the outer membrane protein STM3031 (Ail/OmpX-like protein) of *S. typhimurium* are largely achieved via increasing AcrD efflux pump activity [113]. In addition, hydrogen peroxide stress enhanced the expression of *ompX* mRNA but not OmpX protein in *S. typhimurium*, showing that *ompX* is post-transcriptionally regulated in response to hydrogen peroxide [114]. Meanwhile, three sRNAs (MicA, CyaR, and OxyS; Figure 5) were required in an Hfq-dependent manner to stabilize the *ompX* mRNA [114].

## 7. Interaction

In a variety of circumstances, more than one Omp works concurrently to contribute to the emergence of antibiotic resistance. By selecting porins with desired transmembrane channel diameter, the altered Omps balance in the setting of OmpC/OmpF strongly regulates β-lactam resistance [115]. Non-specific porins appear to be more important in maintaining membrane integrity for susceptibility to non-β-lactam antibiotics, but drug transport by non-specific porins, particularly OmpC and OmpF, considerably influences susceptibility to most β-lactam antibiotics [5]. OmpC or OmpF expression was found in 6.6% of the isolates of carbapenem-resistant *E. cloacae*, with OmpC and OmpF co-expressed in four isolates [116]. The downregulation of OmpF and/or the polarization of the OM transcriptome balance sloped toward the *ompC* gene contribute to carbapenem resistance in *Enterobacter* isolates [117]. Meanwhile, it assumed that this transient polarization of OM protein balance was induced by the phosphorylated CpxR and activated CpxA [118]. The majority of carbapenem-resistant *E. cloacae* isolates displayed decreased membrane permeability due to low *ompC* or *ompF* expression, or both [119]. These results supported earlier studies that suggested reduced *ompF* expression contributes to OmpC-directed OM protein polarization [117]. OmpF and OmpC as well as OmpN porin channels enhance kanamycin absorption into *E. coli* through a size-constricted pore that combines the electrostatic compensation of a steric barrier, which was demonstrated as follows. The small open pore of OmpN does not allow the kanamycin translocation; while the higher negative charge of OmpC is sufficient to compensate for the smaller size of OmpN. Finally, the biggest OmpF pore exhibits decreased flux as a result of an extra binding site being present close to the channel mouth [120]. Additionally, the duration of treatment with antimicrobials and bacterial cells caused various types of porins to react in various ways. In *Yersinia pseudotuberculosis*, the main porins OmpF and OmpC are implicated in the early response to diverse antibiotic stressors caused by sublethal doses. Depending on the antibiotics, they either suppress the transcription level of one or both of their genes, providing the cells with the initial line of resistance. The expression of these two proteins returned to the untreated cells after prolonged antibiotic exposure, despite an increase in the transcription of the alternative porin gene *ompX* [121]. Ceftazidime works well against *E. coli* that produces OmpF, but less well against cells that express OmpC [122].

In addition to OmpC and OmpF’s interaction, additional Omps also showed some sort of association with one another in the resistance process. Overexpressing OmpX in strains of *E. coli* and *E. aerogenes* decreased the expression of non-specific OmpC and OmpF porins, resulting in limited permeability of β-lactams [106,123]. In a nalidixic acid-resistant *E. coli* isolate, Lin et al., observed upregulation of OmpC and OmpW with concurrent downregulation of OmpF [81]. OmpA and OmpC but not OmpF play significant roles in maintaining membrane integrity. Therefore, *E. coli* expresses these three non-specific porins differently, following their diverse functions in preserving membrane integrity [6]. The majority of the linked Omps in *Aeromonas hydrophila* were found to be involved in a complicated protein–protein interaction (PPI) network, which raises the possibility that additional Omps take in the biological roles of the deleted Omps [124]. Meanwhile, among the environmental isolates of *Enterobacter* spp., a relationship between Omp (including OmpA, OmpC, OmpF, and OmpX)-positive isolates and antibiotic resistance (including β-lactam and cephalosporins) was discovered using linear regression performed by GraphPad Prism (v. 7.0) software [125]. In the presence of ceftriaxone, the expressions of *ompA* and *ompX* were found to be low, followed by time- and dose-dependent responses [126]. OmpA and OmpW were considerably downregulated in response to benzyl isothiocyanate (BITC) in *Vibrio parahaemolyticus*, which was explained by the stable complex that BITC and these two Omps generated [127]. In addition to traditional antibiotics, Omps has also been linked to resistance to hostile environments, peptides, natural plant extracts, and nanoparticles. The increased expression of *ompF* and the porosity of *Salmonella*’s outer membrane caused by the deficiency of *ompA* rendered this strain vulnerable to nitrosative stress both in vitro and in vivo [128]. Following incubation with a well-known antimicrobial peptide, Maganin-2 (Mag-2), it was found that the number of OmpA and OmpF generated in *E. coli* gradually decreased [129]. The relative expressions of OmpA, OmpF, and OmpX were noticeably enhanced when *S. enteritidis* was treated with cinnamon essential oil [130]. Through the induction of protein aggregation and electrostatic forces, nanoparticles (NPs) were also utilized as antimicrobial agents, and their treatment of *E. coli* would block the function of OmpA and OmpC [131].

Numerous regulatory mechanisms or signal channels also had an impact on the degree to which Omps were able to coordinate their functions (Figure 1, Figure 2, Figure 3, Figure 4 and Figure 5). The synthesis of the two primary non-specific porins, OmpC and OmpF, in *E. cloacae* isolates diminished or lacked as a result of point mutations that affected their transcription, translation, or insertion into the outer membrane [132]. By sensing and responding to external antimicrobials, as well as controlling the Omps expression levels, two-component systems are typically involved in the development of antibiotic resistance in bacteria. In *Enterobacter* isolates, OmpC and OmpF significantly increased β-lactam and cephalosporin resistance, which was partially controlled by CpxAR and EnvZ/OmpR [82]. The sensor kinase CpxA detects peptidoglycan breakdown when *Klebsiella aerogenes* is exposed to β-lactams, and the response regulator CpxR subsequently represses *ompF* and activates *acrD*, which encodes the efflux pump, resulting in antibiotic resistance [133]. By affecting the expression levels of *ompC*, *ompF*, and *ompW*, a two-component regulator, CpxR, contributes to *S. typhimurium* resistance to aminoglycosides and β-lactams, implying that many Omps work together to exhibit their resistant properties collectively under the control of CpxR [134]. The two-component system BaeSR contributes to the antibiotic resistance of *E. coli*, and BaeR overproduction can reduce cephalosporin susceptibility in the multidrug efflux pump *acrB*-free *E. coli* by lowering the expression level of outer membrane proteins such as OmpA, OmpC, OmpF, OmpW, and OmpX, which is associated with the reduction in TolC and global transcriptional regulators MarA and Rob [135]. The multiple mutations within the CA domain region of EnvZ, a prototypical sensor histidine kinase, and altered ATPase activity of this enzyme were responsible for the expressional changes of OmpC and OmpF porins, which alter the permeability of *Salmonella*’s membrane, boost cefotaxime tolerance, and increase minimal resistance to various classes of antibiotics [136]. Through the control of cell membrane permeability and efflux pump activity, CpxAR and PhoPQ were significant contributors to the emergence of multidrug resistance (MDR) in *S. enteritidis*. This was accomplished in part by enhanced expression of the membrane porin genes, *ompC* and *ompF*, as well as other Omps [137]. Meanwhile, MzrA, a type II membrane protein, connects the two-component envelope stress response regulators, CpxA/CpxR and EnvZ/OmpR. The activated CpxA/CpxR triggers the synthesis of MzrA, which modulates EnvZ/OmpR’s activity through the interaction with EnvZ to favor the accumulation of OmpR~P. OmpC expression is upregulated by high amounts of OmpR~P; however, OmpF expression is downregulated. High OmpR~P also downregulates *mzrA*, creating a negative regulatory feedback cycle [138]. In addition, the loss of *pfs*, a 5′-methylthioadenosine/S-adenosylhomocysteine nucleosidase, greatly enhanced the transcription levels of OmpC and OmpF, as well as the outer membrane permeability of avian pathogenic *E. coli*; however, the transcription levels of the efflux pump gene *tolC* were significantly reduced, which implies that Pfs is a bond that connects Omps and efflux proteins [139]. In *E. coli*, premature stop codons or gene interruptions were shown to cause a decrease in OmpC and/or OmpF, which was associated with meropenem resistance [140].

## 8. Conclusions

In their OM, gram-negative bacteria have different porins (OmpA, OmpC, OmpF, OmpW, and OmpX) with different weights and structures. However, a basic β-barrel was harbored by all the porins mentioned here. Some of these porins will be activated in the presence of different antimicrobial agents to protect the bacterial cells from being killed or inhibited in a strain- and antimicrobial-dependent manner (Table 1). In addition, it has been found that the activity and function of several porins were regulated by a great number of other proteins or regulatory factors (Figure 1, Figure 2, Figure 3, Figure 4 and Figure 5), such as two-component systems (BaeSR, EnvZ/OmpR, and CpxAR), sRNAs (MicA, MicC, MicF, CyaR, and OxyS), SmpB, BlsA, UxuR, H-NS, SoxS, and EmrE.

**Table 1:** Structural features and mediated antibiotic spectrum of typical porins in gram-negative bacteria.

Name	Structural Feature	Drug Resistance Spectrum
OmpA	β-barrel-shaped monomeric protein [26]	Cephalothin, cephaloridine, nalidixic acid, chloramphenicol, trimethoprim, aztreonam, imipenem, colistin, and meropenem [31,32,33,34,35]; β-lactams, amphenicols, glycopeptides, and licosamides [6]; and ceftazidime and meropenem [38].
OmpC	Sixteen-stranded β-barrels [50,73]	Imipenem [53,54]; carbapenem [55]; cefotaxime [52]; and streptomycin, fusidic acid, nitrofurantoin, carbapenems, cefepime, carbapenems, fourth-generation cephalosporins, imipenem, vancomycin, and furomycin [6,56,57].
OmpF	Sixteen-stranded antiparallel β-barrel in tight homotrimers [63]	β-lactams and fluoroquinolones [6,50]; β-lactam antibiotics, including ampicillin and cefoxitin [6,71,72,73,74,75,76]; enrofloxacin [63]; quinolone [77]; nitrofurantoin [80]; and tetracycline [78,79].
OmpW	Eight-stranded β-barrel with a hydrophobic channel [86]	Nalidixic acid [81]; kanamycin, colistin/carbapenem, and ceftriaxone [86,88]; ampicillin, tetracycline, and ceftriaxone [90]; enrofloxacin [91]; tobramycin [92]; ciprofloxacin and imipenem [93]; colicin S4, chlortetracycline, neomycin, and ampicillin [94,95,96]; methyl viologen [97]; and penicillin, kanamycin, and polymyxin B [98].
OmpX	Eight-stranded antiparallel β-barrel [108]	Amikacin, novobiocin, cephalothin, sulfonamides, gentamicin, and nalidixic acid [80]; tetracycline, ciprofloxacin, chloramphenicol, lincomycin, rifampicin, aminoglycosides (kanamycin and streptomycin), and β-lactam (ampicillin, carbenicillin, ceftazidime, and imipenem) [110]; and β-lactam [111].

In general, antimicrobial exposure can cause several Omps to activate simultaneously in some circumstances, and various Omps are involved in different antibiotic transport or membrane permeability. Non-specific porins are involved in both membrane permeability and antibiotic transport, whereas specific porins are only connected to antibiotic passive transport. This comparison emphasizes how general-purpose porins are functionally versatile. Thoroughly exploiting the structures, functions, and regulatory mechanisms of porins and their relationships would pave the way for the control of gram-negative bacteria using porins as targets.

## Data Availability

Not applicable.
